# Lipid-Reactive T Cells in Immunological Disorders of the Lung

**DOI:** 10.3389/fimmu.2018.02205

**Published:** 2018-09-26

**Authors:** Seungwon Ryu, Joon Seok Park, Hye Young Kim, Ji Hyung Kim

**Affiliations:** ^1^Department of Biomedical Sciences, Seoul National University College of Medicine, Seoul, South Korea; ^2^Institute of Allergy and Clinical Immunology, Seoul National University Medical Research Center, Seoul, South Korea; ^3^Department of Microbiology and Immunobiology, Harvard Medical School, Boston, MA, United States; ^4^College of Life Sciences and Biotechnology, Korea University, Seoul, South Korea

**Keywords:** pulmonary disorders, lipid antigens, CD1 molecules, CD1-restricted T cells, natural killer T cells

## Abstract

Regulation of T cell-mediated immunity in the lungs is critical for prevention of immune-related lung disorders and for host protection from pathogens. While the prevalent view of pulmonary T cell responses is based on peptide recognition by antigen receptors, called T cell receptors (TCR), on the T cell surface in the context of classical major histocompatibility complex (MHC) molecules, novel pathways involving the presentation of lipid antigens by cluster of differentiation 1 (CD1) molecules to lipid-reactive T cells are emerging as key players in pulmonary immune system. Whereas, genetic conservation of group II CD1 (CD1d) in mouse and human genomes facilitated numerous *in vivo* studies of CD1d-restricted invariant natural killer T (*i*NKT) cells in lung diseases, the recent development of human CD1-transgenic mice has made it possible to examine the physiological roles of group I CD1 (CD1a-c) molecules in lung immunity. Here, we discuss current understanding of the biology of CD1-reactive T cells with a specific focus on their roles in several pulmonary disorders.

## Introduction

The respiratory system comprises the airways and lungs; as such, it is exposed continuously to foreign materials. Consequently, the immune system in the respiratory system experiences both a huge load and a great variety of foreign antigens. This is significant because this exposure can induce immune reactions that damage lung tissue, thereby transiently or permanently impairing vital respiratory functions. The unique exposure profile of the lung has led to the evolution of immune responses that are not observed in lymphoid organs (1). For example, the surface layer of the lung is composed of airway epithelium, which is a specialized tissue that bears cilia. The epithelial cells secrete mucus that traps inhaled foreign materials; the cilia then move the mucus out of the respiratory system. The lung also harbors a considerable number of innate immune cells, including alveolar macrophages, interstitial macrophages, dendritic cells (DCs), and innate lymphoid cells, all of which act as first line defenders and activate the adaptive immune system, namely, B and T cells. T cells play a particularly important role in controlling immune responses associated with various lung diseases.

Classical T cells recognize foreign or self-antigens complexed with major histocompatibility complex (MHC) I and II molecules on the surface of antigen-presenting cells. When these complexes are recognized by T cell receptors (TCRs), the T cells differentiate into effector cells capable of secreting inflammatory cytokines and cytotoxic proteins that orchestrate downstream immune responses. The role of classical T cells in respiratory health and disease has been well studied [Chen and Kolls published a review on these cells (2)]. However, recent evidence suggests that unconventional subsets of T cells that react to lipid antigens presented

on cluster of differentiation 1 (CD1) molecules also play critical roles in pulmonary immune responses. While the percentage of CD1-reactive T cells in the lung is unclear, a previous study reports a significant percentage of these cells among circulating TCRαβ^+^ cells (7% CD4^+^ and 0.2% CD4^−^CD8^−^) within human PBMC population (3).

While CD1 proteins are expressed in every mammalian species that was examined, humans express five isoforms of CD1, categorized as group 1 molecules (CD1a, CD1b, and CD1c), group 2 molecules (CD1d), and group 3 molecules (CD1e) (4–7). All CD1 molecules, apart from CD1e, display lipid antigens within their hydrophobic antigen-binding groove. The T cells that recognize lipids bound to group 2 CD1 (CD1d) molecules are known as natural killer T (NKT) cells; these are the best studied among the CD1-restricted T cells. This reflects the fact that, of all the CD1 molecules, only the *CD1d* gene is conserved in mice and humans: mice lack the other four CD1 isoforms. The presence of CD1d in mice greatly facilitated research on NKT cells, which has shown that these cells play pivotal roles in development of asthma, pulmonary infection, fibrosis, and other pulmonary disorders. NKT cells represent 5–10% of all T cells in the lungs of adult mice (8). Of the *i*NKT cells in the lung, 0.08% of CD45^+^ cells are present in the lung parenchyma and 0.2% are present in the lung vasculature (9). By contrast, T cells in the lungs that are restricted by the group 1 CD1 molecules (CD1a–c) are less well studied. However, a number of recent studies suggest that these T cells also participate actively in immune responses in barrier organs such as the skin and the lungs.

T cells are particularly promising targets in terms of treatments for various lung diseases (2). To facilitate development of these therapies, a better understanding of lipid-reactive T cells is required. To address this, this review will describe the general features of lipid-reactive T cells and the roles they play in development of various pulmonary disorders, including asthma, chronic obstructive pulmonary disorder (COPD), fibrosis, infection, and cancer.

## The CD1 family of lipid antigen-presenting molecules

Classical MHC class I and II molecules present peptide antigens to T cells. By contrast, CD1 molecules present hydrophobic antigens such as lipids to T cells (9). Structurally, CD1 molecules resemble MHC class I molecules in that they comprise an α-chain bearing three domains (α-1, α-2, and α-3), which is bound non-covalently to β2-microglobulin (β2m). However, CD1 molecules have some unique characteristics that distinguish them from MHC class I molecules. First, human *CD1* genes are located on chromosome 1, whereas human *MHC* genes are located on chromosome 6 (in mice, *CD1* genes are on chromosome 3 and *MHC* genes are on chromosome 17) (7). Second, CD1 molecules are less polymorphic. Third, MHC I and II molecules have six pockets in their antigen-binding groove (denoted A–F) whereas the binding groove of CD1 molecules harbors at least two antigen-binding pockets, named A′ and F′; however, these pockets are narrower and deeper than the A–F pockets in MHC molecules. In addition, these pockets are enriched in hydrophobic residues, which aid the stable binding of lipids to the CD1 groove. The subfamilies of CD1 molecules differ in terms of the size (volume and shape) and properties of these antigen-binding pockets. As a result, the CD1 molecules as a group can present a variety of hydrophobic antigens to T cells (10). Various foreign- and self- antigens that react with CD1-reactive T cells have been identified. These antigens include lipids, phospholipids, glycolipids, and lipopeptides with a large spectrum of size and polarity (10). In general, the hydrocarbon tails, usually alkyl chains, of lipids are buried in the pocket of CD1 molecules and the polar portions protrude, thereby providing a template for TCR engagement (11). Recent studies suggest that many lipid ligands of CD1 molecules, especially CD1c, do not interact directly with TCR; rather they affect the interaction between the TCR and CD1 molecules, thereby allowing or blocking activation of autoreactive T cells (11, 12).

All CD1 isoforms, except CD1e, present antigen. Mice express only CD1d (13), while other mammals (ranging from alpacas to sloths) harbor different combinations of the five CD1 isoforms [these are summarized in the Table 1 from (7)]. CD1e participates in presentation of lipid antigens only indirectly: it trims and transfers lipid antigens prior to presentation to other CD1 molecules (14, 15). CD1a-c molecules are expressed by professional antigen-presenting cells and thymocytes. In particular, Langerhans cells prominently express CD1a while DCs express CD1b and marginal zone B cells express CD1c. The group 2 CD1 molecule, CD1d, is expressed by both hematopoietic and non-hematopoietic cells in various organs, including skin, liver, and colon (16, 17). These differential expression patterns of CD1 molecules suggest that the individual CD1 isoforms may shape local T cell responses by presenting tissue-specific lipids. Moreover, when blood monocytes and hematopoietic CD34^+^ progenitor cells are cultured with granulocyte-macrophage colony stimulating factor (GM-CSF) and IL-4, CD1a expression is induced (18–21). This suggests that CD1 expression can be controlled as necessary *in vivo*.

CD1 molecules load and present lipid antigens in a manner distinct from that of MHC molecules (22, 23). Initially, they are loaded with self-antigen immediately after synthesis in the endoplasmic reticulum (ER); this process is aided by a lipid-transfer protein (LTP) called microsomal triglyceride transfer protein (24, 25). The CD1 molecules then undergo intracellular trafficking (like other proteins synthesized in the ER). Thus, they enter the secretory pathway, which traffics them from the ER to the Golgi apparatus and then finally to the plasma membrane. Sometimes, CD1 molecules exchange their self-antigen for an exogenous lipid antigen at either the plasma membrane or in various endosomal compartments, including early and late endosomes and lysosomes (26). Different CD1 molecules preferentially enter different endosomal compartments due to their disparate sorting motifs. For example, CD1b bears the AP3 sorting motif, which promotes entry into lysosomes (17). In addition, CD1a molecules lack sorting motifs and are therefore recycled to early endocytic compartments such as Birbeck granules, a specialized organelle found in Langerhans cells. Trafficking of CD1a molecules to this compartment is important for lipid antigen presentation to CD1a-restricted T cells (27). These disparate trafficking pathways suggest that different CD1 molecules capture distinct subsets of foreign antigens. In the endosomal compartments, capture of exogenous lipids is mediated by LTPs, including the CD1e molecule. For example, the self-lipid antigens expressed on CD1d molecules are removed and replaced by foreign lipid antigens with the help of a lysosomal LTP called saposin (28, 29). It should be noted that while some CD1 molecules retain the self-antigen that they received at the ER, they can also be loaded with another self-antigen. These CD1: self-antigen complexes can be recognized by autoreactive T cells. The original self-antigen can also function as a chaperone that promotes CD1 stability and trafficking to the plasma membrane, or as a determinant of activation of autoreactive T cell activation (12, 17).

### Lipid-reactive T cells

#### Group 1 CD1-restricted T cells

Research on group 1 CD1 molecules (CD1a-c) is limited by the fact that mice lack homologs of these proteins. Therefore, most studies have been conducted *in vitro* using human cells. T cells that recognize CD1a-c are more common in human peripheral blood than T cells that recognize CD1d: ~2%, ~1%, and ~7% of αβTCR^+^ cells in human peripheral blood recognize CD1a, CD1b, and CD1c, respectively, whereas only ~0.1% of αβTCR^+^ cells recognize CD1d (3, 30). This indicates the need for further studies on the functions of T cells that are restricted by CD1a-c, even though there are some discrepancies regarding their percentages in different models/individuals.

Difficulties associated with studying CD1a-c-restricted T cells can be overcome by using a humanized CD1 transgenic mouse model (hCD1Tg) (31, 32) or humanized SCID mice that have been engrafted with human thymus, liver, and CD34^+^ hematopoietic cells (33). Felio et al. used hCD1Tg mice to examine responses of CD1a-c-restricted T cells to *Mycobacterium tuberculosis* (Mtb) infection. They showed that Mtb-responsive CD1a-c-restricted T cells did not respond quickly to the infection; rather, they became activated later. Moreover, upon second stimulation, they showed boosted responses. Thus, they do not have the innate immune cell-like activities of NKT cells, which are restricted by CD1d and exhibit strong early responses; rather, they more closely resemble classical adaptive lymphocytes (31). This finding was validated by de Lalla et al., who showed that CD1a-c-restricted and self-reactive T cells within adult PBMCs are more likely to be memory T cells than are the same cell populations in umbilical cord blood (3). However, CD1a-c-restricted T cells do not differ from CD1d-restricted T cells in terms of their ability to secrete Th1/Th17 (31, 34) and Th2 (35, 36) cytokines. The percentage and number of CD1d-restricted T cells in the mouse spleen increase with age (37). However, in human blood, age is inversely related with the frequency of CD1d-restricted T cells (38), suggesting that aging-associated accumulation of NKT cells may depend on the species or environment. Classical T cells also exhibit age-related changes in memory phenotype (3).

Although our understanding of development of CD1a-c-restricted T cells remains poor, Li et al. followed the development of HJ1, a human T cell clone restricted by CD1b; they did this by creating a double transgenic mouse that expressed the TCR of the clone and human CD1b (HJ1Tg/hCD1Tg mice) (34). They showed that to develop properly, HJ1 T cells must encounter CD1b-expressing hematopoietic cells in the thymus. In the absence of CD1b-mediated thymic selection, most HJ1 T cells remained at the CD4^+^CD8^+^ double-positive (DP) stage and did not undergo positive selection. However, if CD1b-expressing hematopoietic cells were present, HJ1 T cells underwent positive and negative selection. During this process, the cells changed from DP cells to DP^dull^ and then to double-negative cells. These changes were accompanied by expression of activation markers CD69, CD122, and CD44. They also expressed PLZF (promyelocytic leukemia zinc finger, which is encoded by *Zbtb16*), as did CD1d-restricted invariant NKT (*i*NKT) cells. These observations suggest that CD1a-c-restricted T cells may develop *via* the same developmental pathway used by *i*NKT cells (34). However, further research into the mechanism by which CD1a-c-restricted T cells develop, and into the endogenous lipids involved in this process, is needed.

#### Group 2 CD1-restricted T cells

Group 2 CD1 (CD1d)-restricted T cells are called NKT cells. They are a small subset of CD1-restricted αβ T cells that express both NK cell markers (NK1.1 in mice and CD161 in humans) and TCRs. They respond quickly during the early phase of immune reactions by secreting cytokines. They also play a critical role in bridging the innate and adaptive immune responses (39).

There are two subsets of NKT cells. Type I NKT cells are referred to as invariant NKT (*i*NKT) cells because they have a semi-invariant TCR that is encoded by the *V*α*14-J*α*18* or *V*α*24-J*α*18* TCRα gene segments in mice and humans, respectively. *i*NKT cells respond strongly to α-galactosylceramide (α-GalCer), a glycolipid ligand first isolated from a marine sponge, when it is presented on CD1d molecules (40). Several other endogenous ligands, including α-linked glycosylceramides (41) and β-linked glycosylceramides (42) stimulate *i*NKT cells. However, the ligands that are responsible for the selection of *i*NKT cells in the thymus remain unclear at present. However, the fact that the β-linked glycosylceramides used for some research turned out to be contaminated with α-linked anomers, which are very potent stimulators of *i*NKT cells, negates some of the findings (41, 43, 44). In addition to α-linked monoglycosylceramides, isoglobotrihexosylceramide (iGb3), a lysosomal glycosphingolipid, is an endogenous ligand for *i*NKT cells (45). Mice deficient in hexosaminidase b (Hexb), which converts iGb4 to iGb3, fail to develop *i*NKT cells, supporting the *in vivo* significance of iGb3 as an endogenous ligand (45). However, iGb3 synthase-deficient mice exhibit normal development of *i*NKT cells (46). Still, given that iGb3 stimulates *i*NKT cells in a TCR-dependent manner, it might be a *bona fide* endogenous ligand. Several exogenous ligands derived from bacteria and fungi have been identified. Glycosylceramides derived from the cell wall of *Sphingomonas*, and glycolipids from *Streptococcus pneumoniae*, activate *i*NKT cells in a CD1d-dependent manner (47–49). In addition to bacterial products, a fungal glycolipid called asperamide B can be presented on CD1d, thereby stimulating both human and mouse *i*NKT cells (50). Interestingly, microbial products can activate *i*NKT cells *via* indirect mechanisms by inducing endogenous glycolipid ligands (51, 52). TLR pathways facilitate biosynthesis of glycosphingolipids, which are endogenous ligands for *i*NKT cells. Of note, cytokines take part in the TLR-mediated *i*NKT cell activation. TLR4 and TLR9 mediate IFN-γ production by *i*NKT cells, not IL-4 production, in IL-12- and type I IFN dependent manners, respectively (51). Such cytokine contributions require TCR engagement for the optimal *i*NKT activation. However, it is also shown that cytokines themselves could activate *i*NKT cells independently to TCR triggers at certain levels (51–53). Moreover, cytokines and antigens are differentially required for cytokine production by *i*NKT cells, depending on the stimulants (54). TLR-induced, not ligand-driven, IFN-γ production by *i*NKT cells is impaired in the absence of IL-12. On the other hand, ligand- or TLR- mediated IL-4 production by *i*NKT cells does not require IL-12. During infections, *i*NKT cells produce IFN-γ in a manner dependent on IL-12 and IL-18; However, expression of IL-4 is independent of IL-12 but dependent on CD1 ligands and MyD88 (54, 55). Interestingly, IL-4 production in *i*NKT cells is prominent under Th2 inflammatory settings found in airway inflammation (56).

**Figure 1:**
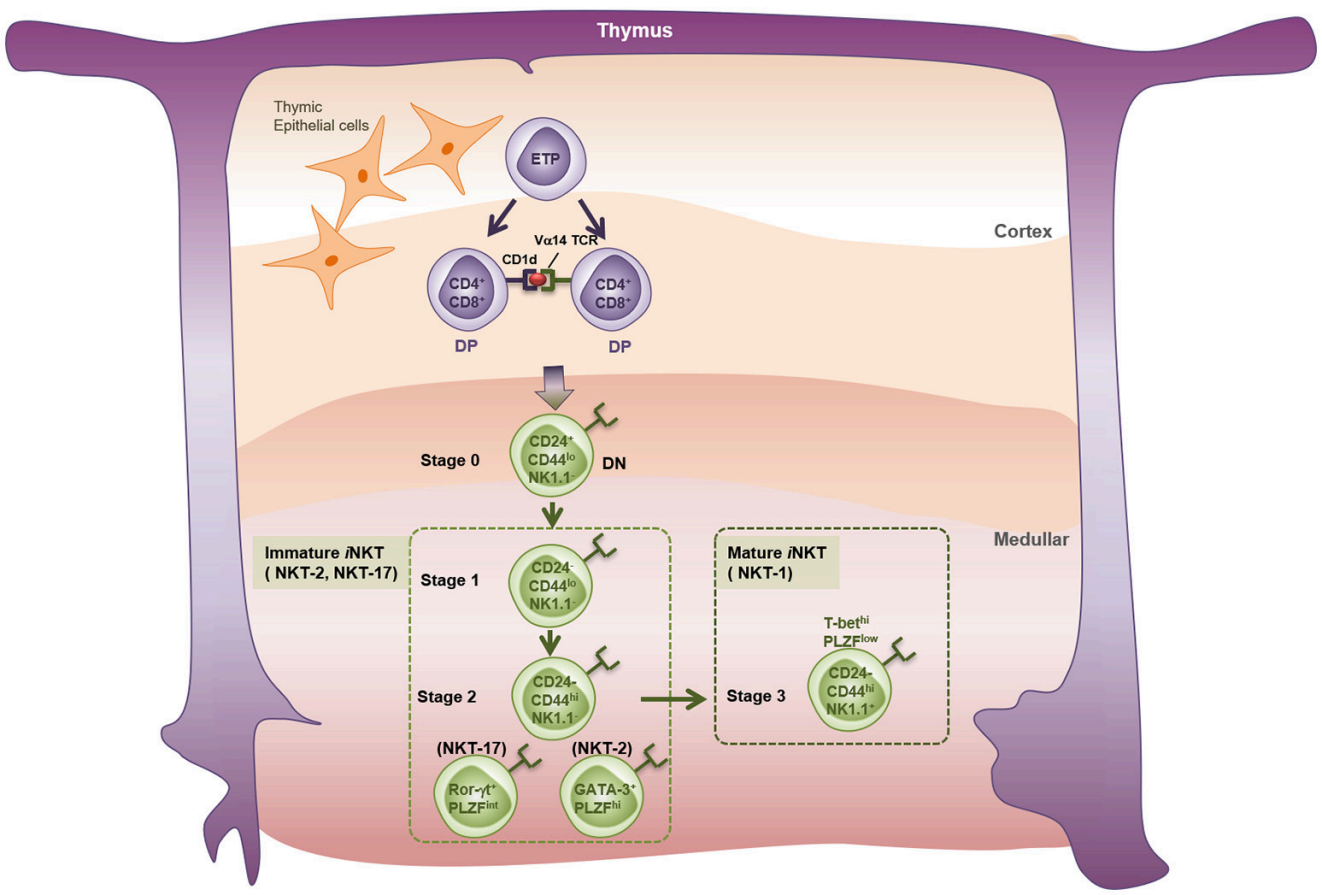
Thymic development of *i*NKT cells. *i*NKT cell precursors originate from the common thymic precursors of conventional T cells. Early T-cell precursors (ETP) give rise to CD4 and CD8 double positive (DP) thymocytes expressing TCRβ chain and rearranged TCRα chains in the thymic cortex. Thymocytes bearing Vα14 TCRs undergo the positive selection by CD1d-expressng DP thymocytes, but not by thymic cortical epithelial cells, and enter the lineage of *i*NKT cells. Endogenous lipid antigens responsible for the development of *i*NKT cells are not fully described. Classically, the thymic *i*NKT cells detected by α-GalCer-loaded CD1d-tetramer are divided into four developmental stages based on their surface markers. Stage 0: CD24^+^CD44^−^NK1.1^−^, Stage 1: CD24^−^CD44^−^NK1.1^−^, Stage 2: CD24^−^CD44^+^NK1.1^−^, Stage 3: CD24^−^CD44^+^NK1.1^+^. A new perspective has categorized thymic *i*NKT cells into three subtypes on the basis of expression patterns of specific transcription factors and cytokines. NKT-1: T-bet^hi^PLZF^lo^ and IFN-γ^+^, NKT-2: GATA-3^+^PLZF^hi^ and IL-4^+^, NKT-17: Rorγt^+^PLZF^inter^, and IL-17^+^.

Despite expressing an invariant TCR, *i*NKT cells are quite heterogenous. When Watarai et al. examined expression of IL17RB (a receptor for IL-25) and CD4 in response to IL-2, they identified three subpopulations of thymic *i*NKT cells; namely, a CD4^+^IL17RB^+^ population that produces Th2 and Th17 cytokines (IL-13, IL-9, IL-10, IL-17A, and IL-22), a CD4^−^IL17RB^+^ population that produces Th17 cytokines (IL-17 and IL-22), and a CD4^+^IL17RB^−^ subset that produces IFN-γ (57). These findings were validated by the study of Lee et al., who showed that NKT cells can be classified as T-bet-expressing NKT1, GATA-3-expressing NKT2, and RORγt-expressing NKT17 lineages (58). Recently, two studies identified new NKT subsets; namely, Bcl-6-expressing follicular helper NKT_FH_ (59) and Nfil3-expressing NKT10 (60, 61) cells. In terms of thymic development (Figure 1), *i*NKT cells develop from a common lymphoid progenitor. Interestingly, however, positive selection of *i*NKT cells is mediated by DP thymocytes (CD4^+^CD8^+^), which present the lipid-CD1d complex, rather than by thymic epithelial cells (62). After positive selection, *i*NKT cells progress from stage 0 to stage 3 (63). Thus, CD24^high^CD44^low^NK1.1^−^ stage 0 NKT precursors first develop into immature stage 1 cells that express CD24^low^; they also express GATA-3 and therefore produce IL-4 (64). Stage 1 cells then become CD24^low^CD44^high^NK1.1^−^ stage 2 NKT cells, which eventually develop into mature CD24^low^CD44^high^NK1.1^+^ stage 3 NKT cells that produce IFN-γ (65–67). PLZF is thought to be the key transcription factor that drives NKT cell differentiation (68, 69). While all NKT cells at all stages express *Zbtb16* (the gene that encodes PLZF), conditional knockout of *Zbtb16* in NKT cells showed that NKT cells cannot develop into immature stage 2 NKT cells without PLZF; mutant mice accumulated stage 1 NKT precursors in the thymus (68). However, considering that NK1.1 is not expressed by all mouse strains, another model of NKT development has been proposed; namely, that the NKT1, NKT2, and NKT17 lineages develop separately from a common NKT precursor rather than by passing sequentially from stage 0 to stage 3 (58, 70).

The second subset of NKT cells, namely, type II NKT cells, express a variable TCR that is restricted by CD1d but is not recognize by α-GalCer (71). Instead, type II NKT cells can be stimulated by sulfatide, β-GalCer, phosphatidylinositol, and phosphatidylglycerol (72). Studies of type II NKT cells are limited by a lack of specific markers for these cells and by a paucity of techniques that permit their analysis (by contrast, *i*NKT cells can be readily and specifically detected using CD1d/α-GalCer tetramers) (73). However, several experimental strategies have enabled study of the functions of type II NKT cells; these include the use of Jα18-deficient IL-4 reporter mice (74) and 24αβ transgenic mice that express the TCR of a type II NKT cell clone (75). These studies showed that, like *i*NKT cells, type II NKT cells develop *via* a linear maturation process marked by changes in expression of CD44 and NK1.1; moreover, like *i*NKT cells, they differentiate into NKT1, NKT2, and NKT17 cells, as shown by their expression of key transcription factors. While these cells remain relatively poorly characterized, it is possible that type II NKT cells may contribute to lung pathology in humans; indeed, evidence suggests that their close cousins, type I NKT cells, play a role in lung disorders (see below for more details). Moreover, humans harbor more type II NKT cells than type I NKT cells (76). Although the CD1-reactive T cell population in the lung has not been analyzed thoroughly, the spatial distribution of different subsets of *i*NKT cells in the mouse lung was investigated using a novel approach aimed at distinguishing *i*NKT cells in lung parenchyma from those in lung vasculature (9). The dominant *i*NKT subset in lung parenchyma was *i*NKT17, whereas that in lung vasculature was *i*NKT1. In both compartments, *i*NKT2 was the least populating subset. Accumulation of pulmonary *i*NKT cells relies on the CXCR6-CXCL16 axis (77). By contrast, intratracheal injection of an α-GalCer analog PBS57 promotes extravasation of CD1d-restricted T cells to the lung parenchyma *via* interaction between CCR4 and CCL17 (8).

## Lipid-reactive T cells in pulmonary disorders

### Asthma

Asthma is a well-known allergic disease of the lung that affects over 7% of adults in the United States of America (78). Symptoms include shortness of breath, coughing, wheezing, and airway hyper-responsiveness (AHR). Asthma can be classified broadly into two phenotypes, allergic and non-allergic, based on production of critical immune mediators. Allergic asthma accounts for more than 50% of asthma cases. It is known as a “Th2 disorder” because type 2 cytokines are pivotal for progression of the disease (79). While conventional Th2 cells contribute to development of allergic asthma, many studies suggest that lipid-reactive NKT cells and innate lymphoid cells may also play a role in this pathology (80, 81).

A variety of studies suggested that CD1d-restricted NKT cells could be critical inducers of asthma in murine models. An ovalbumin (OVA)-induced model of asthma first showed that mice lacking *i*NKT cells (i.e., CD1d- or Vα14-Jα18-deficient mice) do not develop AHR, which is a critical feature of asthma (82–85). Mechanistically, IL-4 and IL-13 which are produced by *i*NKT cells, are essential for development of OVA-induced AHR (83). Other asthma-related features, including airway eosinophilia, the elevation of both type 2 cytokine levels in bronchoalveolar lavage fluid (BALF), and allergen-specific IgE levels in serum, are also dependent on *i*NKT cells. Moreover, *i*NKT cells induce asthma even when CD4^+^ T cells are absent; when MHC II-deficient mice (which lack conventional CD4^+^ T cells but have *i*NKT cells) were challenged with α-GalCer, the mice developed AHR equally as well as the wild-type mice (86). The contribution of *i*NKT cells to development of asthma has also been tested with respect to real-world allergens (rather than OVA or α-GalCer, which are experimental triggers). *i*NKT-deficient mice develop less allergic asthma compared to wild-type mice when exposed to ragweed (87), house dust extracts (88), or a fungus (*Aspergillus fumigatus*) (50). In addition, Pichavant et al. showed that *i*NKT cells expressing IL-17A are essential for development of non-allergic asthma induced by exposure to ozone, another real-world cause of asthma (89).

However, studies by other groups suggest that *i*NKT cells are not required for allergic airway inflammation characterized by eosinophilia and type 2 cytokine production (90–93). These studies show that airway inflammation is dependent on MHC class II molecules, not on CD1d molecules (90, 92). Also, β2m-deficient mice, which are unable to express CD1d molecules, develop asthma despite lacking *i*NKT cells (91, 93, 94). However, Koh et al. suggested that some NKT cells in β2m-deficient mice are restricted to a β2m-independent form of CD1d, and these cells can compensate for deficiency of conventional *i*NKT cells. Despite this possibility, *i*NKT cells might become more dispensable than T cells with respect to inducing AHR when mice are challenged repeatedly with antigens such as OVA (90, 92).

It should be noted that Jα18 (or *Traj18*) knockout mice, which are often used to study the roles of *i*NKT cells and type II NKT cells, show a significant (>60%) reduction in TCRα chain diversity (95). A recent study attempted to resolve this issue by developing a new type of *Traj18* knockout mouse strain lacking *i*NKT cells but with normal TCRα diversity. When OVA- and cockroach allergen-induced asthma were assessed in these mice, knockout mice showed significantly less severe AHR than wild-type mice (96). Taken together, the results from murine models of asthma are conflicting; however, *i*NKT cells may contribute to the pathogenesis of asthma under certain conditions.

Akbari et al. (97) conducted flow cytometry experiments and found that BALF from patients with moderate to severe asthma contained a very high percentage of *i*NKT cells (about 60% of all CD3^+^ cells); BALF from healthy subjects contained very few *i*NKT cells (97). It should be noted, however, that several other groups found low percentages (<2%) of *i*NKT cells in BALF from adult asthmatics; these percentages did not differ significantly from those in control subjects detected using CD1d tetramers, an anti-Vα24 antibody, or an anti-Vβ11 antibody (98–101). It was proposed that differences in the percentage of *i*NKT cells in BALF, sputum, or bronchial biopsies may be due to nonspecific binding of CD1d-tetramers, or to inappropriate gating on *i*NKT cells (102). Other human studies noted increased percentages of *i*NKT cells in BALF from childhood asthma patients (0.435% of αβ T cells in asthma vs. 0.116% of αβ T cells in control subjects) (103), and in the lungs of patients with severe asthma compared with patients with well controlled asthma (104). Thus, the link between the percentage of *i*NKT cells in human lung or bronchial specimens from asthma patients and disease pathogenesis remains still unclear.

A functional study from the United Kingdom, which was based on a human allergen challenge model of asthma, revealed that the percentage of *i*NKT cells in bronchial biopsies taken 24 h after allergen challenge increased by 15% along with the increase in AHR (105). Moreover, Agea et al. showed that the PBMCs from subjects who were allergic to cypress pollen responded to two specific lipid antigens in the pollen, namely, phosphatidylcholine and phosphatidylethanolamine. These lipid antigens, which are loaded onto CD1 molecules, stimulated T cells within the PBMCs in CD1a- or CD1d-dependent fashion (106). Thus, both CD1a- and CD1d-restricted T cells may participate in allergic reactions in humans.

Although most studies to date have focused on the pathogenic roles of *i*NKT cells in asthma, *i*NKT cells also have protective functions under specific preconditions. For example, when young mice are infected with influenza A virus, they are protected against allergen-induced AHR when they become adults (107). CD4^−^CD8^−^ DN NKT cells showing high expression of CD38 produce IFN-γ and induce CD4 T cell proliferation in a contact dependent manner (108). Glycolipids from *Helicobacter pylori* activate suppressive NKT cells (107). Notably, Sharma et al. showed that benzo[a]pyrene, a classical environmental air pollutant that causes respiratory disease, suppressed IFN-γ production by CD1a- and CD1d-restricted T cells *in vitro* by downregulating expression of CD1a and CD1d by human DCs (109). Thus, it appears that certain subsets of NKT cells, such as IFN-γ-producing NKT cells, may suppress AHR.

Because research into the roles of CD1d-restricted *i*NKT cells in asthma has produced conflicting results, comprehensive studies aimed at characterizing the cause and identifying the contribution made by these cells are needed. Also, we should not overlook the fact that *i*NKT cells alter the cytokine milieu and affect asthmatic responses under certain conditions. Further studies should determine the role of CD1a-c restricted T cells in the pathogenesis of asthma.

### Chronic obstructive pulmonary disease (COPD)

COPD is another chronic pulmonary disease that causes difficulty in breathing; however, it differs from asthma in several aspects. First, it is not an allergic disease. Second, the airway obstruction is largely irreversible. Third, smoking is a well-known risk factor for COPD. Fourth, it is characterized pathologically by either chronic obstructive bronchiolitis or emphysema (110). Although one study shows that the percentage of *i*NKT cells in induced sputum from COPD patients are not different compared to that in control subjects (101), others suggest that *i*NKT cells contribute to the pathogenesis of COPD. For example, mice with chronic lung disease caused by Sendai virus infection harbor large numbers of alternatively-activated IL-13-secreting macrophages in the lung. Experiments with CD1d or Jα18 knockout mice revealed that *i*NKT cells are required to generate virus-induced IL-13-producing macrophages. Thus, *i*NKT cells facilitate chronic airway inflammation in mice (111). The same authors showed that a similar mechanism may also participate in COPD. Immunofluorescence staining of lung tissues from patients with COPD and from normal controls revealed that the former harbored significantly higher numbers of Vα24^+^ *i*NKT cells and IL-13^+^CD68^+^ macrophages (111). Wang et al. also showed that patients with COPD harbor higher percentages of CD56^+^CD3^+^ NKT cells in the blood than control subjects (although these data should be interpreted carefully due to the possibility of confounders, i.e., smoking, with respect to NKT cells and COPD) (112). In addition, Pichavant et al. found that oxidative stress generated by cigarette smoke activates DCs and airway epithelial cells, which in turn cause *i*NKT cells to accumulate in the lung and release IL-17 (113). Furthermore, when mice are challenged repeatedly with intranasal α-GalCer, *i*NKT cells are activated and the mice develop COPD-like symptoms, including mucus hypersecretion and pulmonary emphysema (114). Taken together, these results suggest that *i*NKT cells might play a pathogenic role in COPD. To date, no study has investigated the role of CD1a-c-restricted T cells in COPD. However, limited number of studies in humans evaluated CD1a expression in DCs from COPD patients (115–117). These studies suggested either no difference or an increase in the number of alveolar langerin-positive and CD1a^+^ immature DCs in response to chronic cigarette smoking, which contribute to the COPD pathology. The role of CD1a-c-restricted T cells in COPD remains unclear.

### Pulmonary fibrosis

Pulmonary fibrosis is a collection of chronic progressive lung diseases characterized by abnormal wound healing, which in turn generates thickened and stiff lung tissue. The causes of lung fibrosis are numerous; however, most studies on the role of *i*NKT cells focused on idiopathic pulmonary fibrosis (IPF). IPF is a chronic, progressive fibrotic lung disease that is diagnosed (when no other cause of fibrosis can be found) according to the results of histology or high-resolution CT. With the exception of the conditionally recommended use of pirfenidone and nintedanib (118), no drugs improve the survival of patients with IPF. Therefore, better understanding of the disease is required (119). A common animal model used to study the pathophysiology of IPF is bleomycin-treated mouse; bleomycin is a chemotherapeutic agent that causes lung fibrosis as a side effect. Jα18 knockout and CD1d knockout bleomycin-treated mice exhibited exacerbated symptoms of lung fibrosis (120). Moreover, treatment with α-GalCer ameliorated lung fibrosis in an *i*NKT cell-dependent manner (121). Of note IFN-γ produced by *i*NKT cells was responsible for *i*NKT-mediated protection against fibrosis (120). However, other studies suggest either that *i*NKT cells are not necessary for lung pathogenesis, or that they play a pathogenic role, when mice are not treated with exogenous ligands (121, 122). Despite this discrepancy with respect to the impact of endogenous ligands or cytokine-driven activation of *i*NKT cells on disease pathogenesis, exogenous ligand-induced activation of *i*NKT cells consistently protects against lung fibrosis (120–122). Thus, *i*NKT cells likely protect against lung fibrosis. In contrast to CD1d-restricted *i*NKT cells, the function of CD1a-c-restricted T cells has not been studied either human or mouse models of pulmonary fibrosis.

### Lung infection (pneumonia)

Pneumonia is an inflammatory lung disease caused by viral, bacterial, or fungal infections (123). Unlike the other pulmonary diseases discussed above, many lines of evidence suggest that group 1 CD1-restricted T cells respond to respiratory infections.

Pulmonary infection with Mtb results in an atypical pneumonia called pulmonary tuberculosis (TB). It is a significant public health concern because TB is a highly contagious airborne disease. Moreover, despite development of the *M. bovis*-derived Bacillus Calmette-Guérin (BCG) vaccine long time ago, it is still difficult to prevent TB in adults. These factors, along with the high prevalence of TB, its disease course, and practical issues regarding long-term treatment regimens, have led to great interest in understanding Mtb infection and in developing new vaccines that will curb TB (124). In 1994, Beckman et al. showed that mycolic acid derived from the Mtb cell wall binds to CD1b (125). Later, Moody et al. found that mannosyl-beta1-phosphodolichol and didehydroxymycobactins, another Mtb lipid and a lipopeptide antigens, activate CD1c- and CD1a- restricted T cells, respectively, as well as peripheral blood cells, from patients with TB (126, 127). The percentages of the group 1 CD1-responding T cells in individuals exposed to Mtb are higher than those in control individuals. Furthermore, they produce IFN-γ responses to lipid antigens derived from Mtb, suggesting protective role against the infection (3, 128). However, group 1 CD1-restrictred T cells produce not only Th1 cytokines, but also Th17 and Th2 cytokines (35, 129, 130). Therefore, further studies should investigate physiological role of Th17 and Th2 cytokine-producing group 1 CD1-restricted T cells in infection. Once the human group 1 CD1 transgenic (hCD1Tg) mice became available, more specific functional studies of CD1-restricted T cells in TB infection have been performed. As mentioned above, Felio et al. showed that, when hCD1Tg mice are infected with Mtb, group 1 CD1-restricted T cells exhibit adaptive cell-type responses, and release IFN-γ at a late time-point after primary exposure to Mtb; however, release is much faster after subsequent exposure (31). Contradictory results were reported by Zhao et al.; When they infected hCD1Tg mice (that were also transgenic for a mycolic acid-specific CD1b-restricted TCR) with TB, they found that CD1b-restricted T cells responded more quickly than Mtb-specific CD4^+^ T cells to Mtb challenge (131). These disparate reaction patterns may reflect differences in the methods used by Felio et al. and Zhao et al. with respect to antigen challenge; the former used intraperitoneal injection of Mtb (31), whereas the latter used intranasal challenge (131). In any case, these studies show that group 1 CD1-restricted T cells participate in immune responses to Mtb. Moreover, peripheral blood from BCG-immunized subjects contains a high percentage of group 1 CD1-restricted CD8 T cells, which react with live BCG-infected DCs (132). Interestingly, when Parlato et al. compared the gene expression patterns of Mtb antigen-challenged DCs from patients with active and latent TB infection, they found that DCs from patients with active TB lacked CD1a and CD1c expression. By contrast, DCs from patients with latent TB exhibited upregulated expression of CD1a. This suggests that, even though group 1 CD1-restricted T cells may help to protect the host from TB by producing IFN-γ, specific CD1a–c-restricted subsets of these cells may respond to the same infection in different ways (133).

Group 2 CD1-restricted T cells (specifically, *i*NKT cells) may be involved in immune responses to Mtb. A mouse study showed that injection of an anti-CD1d antibody exacerbated the symptoms of TB, which suggests that *i*NKT cells may have anti-microbial functions (134). In addition, Sada-Ovalle et al. found that adoptive transfer of *i*NKT cells reduced the bacterial burden in Mtb-infected mice by producing IFN-γ and killing intracellular bacteria (55). This protective function of *i*NKT cells suggests that vaccination with common lipid antigens may boost the beneficial anti-TB functions of CD1-restricted T cells (135).

Since human immune responses differ according to the pathogen encountered, it is possible that lipid-reactive T cells respond quite differently to infectious agent other than Mtb. Nevertheless, it is noteworthy that various studies show that *i*NKT cells also largely play protective roles against other pathogens such as *Streptococcus pneumoniae* and *Saccharopolyspora rectivirgula*, the most common causes of community-acquired pneumonia (48, 136) and Farmer's lung (137) respectively. Exceptions include a few cases of non-common pneumonia occurring in adults. For example, the causative agent of atypical pneumonia is *Chlamydia trachomatis*. Patients become infected with *C. trachomatis* either after sexual activity or by vertical transmission from an infected mother. Bilenki et al. showed that, when a mouse model of chlamydial pneumonia is injected with α-GalCer and then infected with *C. muridarum*, the α-GalCer-activated NKT cells increase the chlamydial burden and worsen the symptoms (138).

Contrary to microbial infections in which CD1-reactive T cells are *mainly* activated through microbial lipid-CD1-dependent mechanisms, viral infections are a good model in which to demonstrate the CD1- or TCR-independent mechanisms underlying CD1-restricted T cell activation. Unfortunately, no study has examined the role of group 1 CD1-restricted T cells in pulmonary viral infection, except during systemic viral infections such as HIV (139), which might cause pulmonary infection. Therefore, we focus here on the biology of *i*NKT cells in viral infection models.

Influenza virus is a representative respiratory virus that causes seasonal flu and pneumonia with high morbidity and mortality (140). Several studies showed that NKT cells control lung inflammation during influenza A virus (IAV) infection through dampening suppression of the virus-specific T cell responses or controlling the IAV-associated lethal inflammation. During IAV (PR8, H1N1) infection, *i*NKT cells suppress myeloid-derived suppressor cells (MDSCs), which contribute to lung pathology and inhibit the activity of the virus-specific immune cells (141). The MDSC suppressive activity was inhibited by *i*NKT cells, which were activated in endogenous lipids:CD1d-dependnet manner. MDSCs derived from Hexβ^−/−^ mice (unable to produce endogenous lipid ligands) failed to activate *i*NKT cells, thereby sustained their suppressive capability. In another study showing a protective role of *i*NKT cells in IAV (Scotland, H3N2) infection, however, the underlying mechanism was not mediated by MDSCs, but by a positive effect of *i*NKT cells on the migration of respiratory dendritic cells to the draining lymph nodes, a phenomenon that favors the expansion of virus-specific CD8^+^ T cells (142). Also, *i*NKT cells suppressed the fatal inflammation by lysing infected inflammatory monocytes (the dominant immune cell in IAV-infected lungs) in a CD1d-dependent fashion (143). In contrast to the CD1d-dependent actions of *i*NKT cells, a cytokine-mediated, CD1d-independent *i*NKT cell activation was shown to limit pulmonary inflammation and a secondary bacterial infection during IAV infection. RORγt^+^ *i*NKT cells (*i*NKT17) secrete a lung-protective cytokine, IL-22, in response to stimulation by IL-1β and IL-23 secreted by IAV-infected DCs (144, 145). Interestingly, the reduction of the burden of secondary bacterial infection (*Streptococcus pneumoniae*) after IAV infection was observed when recombinant IL-22 was treated intranasally (146). This new strategy draws an attention considering the severe consequences from the co-infections (147, 148). Although we could speculate that there is no lipid antigen directly from viral genome in cells with virus infection, it should be verified with the *in vivo* system which enabled to distinguish the origin of effector functions (either TCR-dependent or -independent manner) in *i*NKT cells. A Nur77^gfp^ reporter system, which expresses GFP in response to TCR stimulation only, was used to show that IFN-γ-producing *i*NKT cells are induced during infection with murine cytomegalovirus (MCMV) without any change in GFP expression (149). It is now anticipated that the previously reported (i.e., not restricted to infection models) effector functions of CD1-restricted T cells will be re-evaluated using novel approaches to better understand the contribution of the TCR to *i*NKT cell activation.

### Lung cancer

The role of lipid-reactive T cells (especially α-GalCer-reactive *i*NKT cells) in tumors began attracting attention when Kobayashi et al. (150) showed that *i*NKT cells play protective roles in a B16 melanoma mouse model (150). Although CD1-reactive T cells are best known for their ability to produce cytokines, *i*NKT cells (like NK cells and cytotoxic T cells) are also cytotoxic; thus, they may be potential targets for therapies aimed at inducing anti-tumor immunity (151). The first *in vivo i*NKT cell-based immunotherapy regimen tested in patients with a variety of cancers, including breast, colon, liver, skin, kidney, prostate, peritoneum, and lung cancer. The study showed that, when *i*NKT cells were activated by treatment with α-GalCer-loaded DCs, they contributed to the tumor suppressive environment and reduced the size of the tumors (152). It is well recognized that *i*NKT cells protect against lung cancers, particularly non-small cell lung cancers (NSCLC), by producing IFN-γ and cytotoxic proteins (153–155). The percentage of NKT cells (CD56^+^ CD3^+^ T cells) in the blood of NSCLC and SCLC (small cell lung cancer) patients is increased compared to healthy controls (156). In agreement with the beneficial role of *i*NKT cells in lung cancer, the numbers of *i*NKT cells were compared following the cancers of differing histologic type and stage. It was found that the number was: (1) lower in adenocarcinoma, which has the worse prognosis than in squamous cell carcinoma (157); (2) lower in stage 1 than in stage IV cancers (156). Another human study showed that the percentage of Vα24 NKT cells in the PBMC of lung cancer patients was 0.01–0.03%; however, patients with recurrent cancers harbored a significantly lower percentage of NKT cells than healthy donors (158). Collectively, the data suggest that more immunosuppressive environments encountered at the later stages of lung cancer reduce the percentage of *i*NKT cells; this may allow the cancer to escape immune surveillance. In line with this idea, one study shows that injecting *in vitro-*expanded autologous *i*NKT cells may be an effective form of *i*NKT cell-based immunotherapy (159).

Several studies have assessed the role of type II NKT cells in tumor immunity. One such study was conducted by Terabe et al., who showed that when CD1d knockout and Jα18 knockout mice were used as CT26 colon carcinoma metastasis to the lungs, both knockouts developed significantly lower numbers of lung tumor nodules, compared to wild-type mice. However, this effect was more pronounced in CD1d knockout mice (which lack both type I and II CD1d-restricted NKT cells) than in Jα18 knockout mice (which lack only type I CD1d-restricted NKT cells). This suggests that type II NKT may suppress tumor metastasis or growth (160). However, evidence suggests that type I NKT cells promote immunity to CT26 cancer cells while type II NKT cells suppress it. Moreover, an absence of type I NKT cells is associated with stronger type II NKT cell-mediated suppression, whereas type II NKT cells appear to downregulate protective tumor immunity mediated by type I NKT cells. These findings suggest that type I and II NKT cells may counter the effects of each other in a balanced manner (161).

## Conclusion and perspectives

Here, we discussed the role of CD1-restricted T cells in lung diseases, including asthma, COPD, pulmonary fibrosis, infection, and cancer. Studies on group 2 CD1-restricted T cells are prolific. Less is known about group 1 CD1-reactive T cells because mice lack group 1 CD1 genes. Nevertheless, the current state of knowledge suggests that, like conventional T cells, group 1 and 2 CD1-restricted T cells can play either a protective role or a pathogenic role in lung disease (Table 1).

**Table 1:** Studies of lipid-reactive T cells in pulmonary disorders.

**Species**	**Description**	**References**
**ASTHMA**		
Human	Cypress pollen-sensitive patients: 1. Blood CD1-reactive T cells were activated by cypress-derived lipids presented by CD1a^+^ or CD1d^+^ APCs 2. Patient cells produced IL-4 and IFN-γ in response to lipids	(106)
Mouse	Lipophilic air pollutant-induced inflammation: Challenge with benzo[a]pyrene or diesel exhaust particles suppressed IFN-γ-producing CD1a- and CD1d-restricted T cells	(109)
Mouse	OVA challenge model: 1. Reduced AHR in CD1d-deficient mice 2. Restoration of AHR upon adoptive transfer of NKT cells to CD1d-deficient mice	(83)
Mouse	OVA challenge model: 1. Reduced AHR in Jα18^−/−^ mice 2. Reduction of the key features of asthma [eosinophilia, high type 2 cytokine (IL-4 and IL-5) production, and high anti-OVA IgE titers] in Jα18^−/−^ mice 3. Restoration of key features upon adoptive transfer of NKT cells	(84)
Mouse	OVA challenge model: 1. Asthmatic phenotype (AHR, eosinophilia, type 2 cytokines) induced only when challenged with OVA and α-GalCer 2. No AHR in CD1d-deficient or MHC II-deficient mice 3. Increased number of *i*NKT cells in BALF	(85)
Mouse	*Sphingomonas* glycolipid challenge: 1. Asthmatic phenotype (AHR, eosinophilia, histology, type 2 cytokine (IL-4 and IL-13) production, and increased serum IgG) induced only by α-GalCer or *Sphingomonas* glycolipid 2. No effect on AHR in MHC II-deficient mice	(86)
Mouse	Ragweed challenge: 1. Reduced eosinophilia, mucus production, IL-4 levels, and serum IgE levels in the airways of CD1-deficient mice 2. Enhancement of the above features upon exposure of WT ragweed-challenged mice to α-GalCer	(87)
Mouse	OVA or cockroach allergen challenge: 1. Reduced IFN-γ and IL-4 production in Traj18-deficient mice challenged with α-GalCer 2. Reduced AHR in Traj18-deficient mice challenged with either OVA or cockroach allergen	(96)
Mouse	Ozone challenge: 1. Ozone-induced AHR abolished in CD1d1^−/−^ and Jα18^−/−^ mice 2. IL-4, IL-13, and IL-17A contribute to ozone-induced AHR	(89)
Human, Mouse	*Aspergillus fumigatus*-derived glycolipid challenge (Human *i*NKT cell line, CD1d1^−/−^ and Jα18^−/−^ mice): 1. Abolition of AHR induced in CD1d1-deficient mice by *A. fumigatus* extract 2. *A. fumigatus*-derived glycolipid presented by CD1d directly activates human and mouse *i*NKT cells	(50)
Human	Asthma patients: 1. *i*NKT cells in BALF: 63% of CD4^+^ cells in asthma vs. < 1% of CD4^+^ cells in controls 2. *i*NKT cells from asthma patients produced IL-4 and IL-13 in response to α-GalCer	(97)
Human	Childhood asthma patients: *i*NKT cells in BALF: 0.435% of αβ T cells in childhood asthma patients vs. 0.116% of αβ T cells in controls	(103)
Human	Asthma patients: Increased numbers of *i*NKT cells in BALF from asthma patients, especially those with severe asthma (severe asthma vs. control: *p* = 0.004; well controlled asthma vs. control: *p* = 0.02)	(104)
Human	Asthma patients (bronchial biopsy): 1. At baseline: 33 cells/mm^2^ of *i*NKT cells (9.8% of CD3^+^ T cells) in asthma vs. none in controls 2. After allergen challenge: 15% increase in number of *i*NKT cells (from 33 to 59 cells/mm^2^), along with decreased FEV_1_ and increased AHR	(105)
Mouse	OVA or HDM challenge: 1. No difference in AHR between WT and CD1d-deficient mice challenged with OVA or HDM 2. No change in the number of NKT cells in the lung after challenge 3. No difference in severity of AHR when anti-CD1d mAb was administered to the OVA-induced asthma model	(162)
Mouse	OVA challenge: OVA-induced allergic airway inflammation, based on histological analysis and total lung cell counts, did not change in CD1d1^−/−^ or β2m^−/−^ mice compared to wild-type	(90)
Human	Asthma patients: 1. *i*NKT cells in BAL fluid and bronchial biopsy: less than 2% of CD3^+^ T cells in asthma patients 2. *i*NKT cells in induced sputum: no difference in number between asthma and healthy controls	(101)
Human	Asthma patients: *i*NKT cells in BAL fluid: 0.37% of CD3^+^ T cells in asthma vs. 0.12% in controls (*p* = 0.4)	(99)
Human	Asthma patients: *i*NKT cells in induced sputum: 0.07% of T cells in asthma vs. 0.06% of T cells in controls (*p* = 0.75)	(98)
Mouse	H3N1 virus infection/OVA challenge: 1. Adoptive transfer of influenza A virus-infected NKT cells to Jα18^−/−^ mice protects them from OVA-induced AHR 2. A *Helicobacter pylori-*derived glycolipid protects from OVA-induced AHR	(107)
Mouse	OVA challenge: 1. Anti-CD1d antibodies abolished OVA-induced AHR in β2m^−/−^ mice 2. α-GalCer treatment failed to induce AHR in β2m^−/−^ mice	(94)
Mouse	OVA challenge: 1. Sulfatide-activated type II NKT cells ameliorated airway inflammation (as assessed by histologic analysis, IL-4 and IL-5 levels, and total cell counts in BALF) 2. Sulfatide reduced the number of IFN-γ/IL-4-producing *i*NKT cells	(163)
**COPD**		
Human, Mouse	COPD patients: 1. Higher percentage of CD56^+^ CD3^+^ NKT cells in peripheral blood from COPD patients than in that from healthy controls 2. Human IFN-γ- or IL-17-producing *i*NKT cells activated by cigarette smoke extract-exposed dendritic cells or airway epithelial cells •[] Cigarette smoke model (mouse): 1. Increased number of lung *i*NKT cells after exposure to cigarette smoke 2. No induction of AHR in CD1d1^−/−^ or Jα18^−/−^ mice	(113)
Mouse	α-GalCer-induced model: 1. Repeated intranasal challenge with α-GalCer induced COPD (characterized by emphysema, mucus production, and fibrosis) 2. COPD phenotype from α-GalCer-induced IL-4-producing *i*NKT cells was ameliorated by anti-IL-4 mAb treatment	(114)
Human, Mouse	COPD patients: High numbers of *i*NKT cells and IL-13^+^ CD68^+^ macrophages in lung tissues from COPD patients Sendai virus infection model: 1. IL-13^+^ macrophages contribute to lung inflammation induced by viral infection 2. Lower number of IL-13^+^ macrophages in CD1d1^−/−^ or Traj18-deficient mice	(111)
Human	COPD patients: Higher number of CD56^+^ CD3^+^ NKT cells in peripheral blood or induced sputum of COPD patients than in those of controls	(112)
Human	COPD patients No difference in frequency of *i*NKT cells in induced sputum between healthy control and COPD patients (either in acute exacerbations or stable status)	(101)
**PULMONARY FIBROSIS**		
Mouse	Bleomycin-induced model: 1. Prolonged survival after α-GalCer treatment; this was reversed in Jα18^−/−^ mice 2. IFN-γ production induced by α-GalCer treatment contributes to reduced MIP2, TGF-β, and CTGF levels	(121)
Mouse	Bleomycin-induced model: 1. Aggravated lung fibrosis (as assessed by histologic analysis, hydroxyproline levels, and survival rate) in CD1d-deficient and Jα18^−/−^ mice 2. Reversal of fibrosis upon adoptive transfer of *i*NKT cells to CD1d-deficient mice 3. IFN-γ-producing *i*NKT cells secrete TGF-β, which contributes to fibrotic changes	(120)
Mouse	Whole thorax irradiation: No difference in fibrotic changes (as evidenced by histologic analysis and BALF differential cell counts) in WT and Jα18^−/−^ mice	(164)
Mouse	Bleomycin-induced model: Reduced fibrosis (as assessed by hydroxyproline and TGF-β levels) in Jα18^−/−^ mice	(122)
**LUNG INFECTION**		
Mouse	Mtb infection model (humanized CD1 Tg/CD1b-restricted, mycolic acid-specific TCR Tg mice): 1. Mtb controlled by IFN-γ-producing CD1b-restricted T cells, which were activated by Mtb-infected DCs 2. CD1b-restricted T cells activated earlier than Mtb-specific CD4^+^ T cells	(131)
Mouse	*Chlamydia muridarum* infection model: 1. Reduced disease activity [as evidenced by decreased weight loss and bacterial burden, and reduced type 2 cytokine (IL-4 and IL-5) levels] in CD1-deficient mice 2. α-GalCer aggravated infection-induced inflammation	(138)
Mouse	Mtb infection: 1. Suppressed bacterial replication reversed in CD1d^−/−^ and Jα18^−/−^ mice 2. IFN-γ-producing *i*NKT cells reduced the bacterial burden in a dose-dependent manner	(55)
Mouse	*Streptococcus pneumoniae* infection: 1. Increased production of IFN-γ by *i*NKT cells from infected mice 2. Increased bacterial burden upon treatment with an anti-CD1d mAb	(48)
Mouse	Mtb infection: 1. Increased bacterial load in β2m-deficient mice 2. Decreased production of cytokines (IL-12, TNF, IFN-γ, and TGF-β) after the treatment with an anti-CD1 mAb	(134)
Mouse	*Streptococcus pneumoniae* infection model: Increased bacterial load and IFN-γ secretion after anti-CD1d mAb treatment	(136)
Mouse	Influenza A virus (PR8 strain) infection: 1. Recovery of decreased survival rate after PR8 infection after adoptive transfer of *i*NKT cells to Jα18^−/−^ mouse 2. Decreased viral titer after adoptive transfer of *i*NKT cells to Jα18^−/−^ mouse	(141)
Mouse	Influenza A virus (H3N2 strain) infection: 1. Increased expression of IL-22 in early time point of IAV infection 2. *i*NKT cells showed the highest fold change of IL-22 expression among other IL-22-producing cells including αβ T cells, γδ T cells and ILCs 3. *IL22* KO mouse showed decreased survival rate with severe pathology and IAV-specific CD8^+^ T cells compared to WT mouse	(144)
**LUNG CANCER**		
Human	Advanced non-small cell lung cancer, recurrent lung cancer patients (Phase I study): 1. Increased numbers of Vα24 NKT cells in peripheral blood of a few patients (3/11 patients) after intravenous injection of α-GalCer-pulsed DCs 2. Increased IFN-γ production by NKT cells in one patient after stimulation with pulsed DCs 3. No progression of disease in 2/11 patients	(153)
Human	Advanced non-small cell lung cancer, recurrent lung cancer (Phase I study): 1. Increased numbers of Vα24 NKT cells in peripheral blood of 2/3 patients after intravenous injection of α-GalCer-activated NKT cells. 2. Increased numbers of IFN-γ-producing NKT cells in all three patients after intravenous injection of α-GalCer-activated NKT cells	(159)
Human	Advanced non-small cell lung cancer or recurrent lung cancer patients who were refractory to current therapy: 1. Increased numbers of IFN-γ-producing cells in 58.8% of patients after injection of IL-2/GM-CSF-cultured PBMCs pulsed with α-GalCer 2. Prolonged median survival time in responders (who showed an increase in the number of IFN-γ-producing cells) (31.9 months in responders vs. 9.7 months in non-responders)	(154)
Mouse	Model of CT26 colon carcinoma metastasis to the lung (Jα18 ^−/−^, CD1d ^−/−^ mice): 1. Reduced number of metastatic lung nodules in groups treated with anti-CD1d 2. No effect in regulating tumor metastasis/surveillance in Jα18 ^−/−^ mice, compared to the effective CD1d ^−/−^ mice	(160)
Human	Lung cancer patients (non-small cell lung cancer, small cell lung cancer): 1. Increased number of CD56^+^ CD3^+^ T cells in peripheral blood from lung cancers (NSCLC and SCLC) compared to healthy controls (2.91% in NSCLC, 2.97% in SCLC, and 1.6% in healthy controls) 2. Increased number of CD56^+^CD3^+^ T cells in Stage I (9.0%) compared to Stage IV (3.8%) NSCLC patients	(156)
Human	Non-small cell lung cancer patients: 1. Higher *i*NKT cell frequency in tumor than blood (*p* = 0.04) and lymph nodes (*p* = 0.02) 2. Higher *i*NKT cell frequency in T4 cancer stage compared to T1 (*p* = 0.03) 3. The highest *i*NKT cell frequency in squamous cell carcinoma and the lowest *i*NKT cell frequency in adenocarcinoma	(157)
Human	Lung cancer patients (including adenocarcinoma, squamous cell carcinoma, and small-cell lung cancer): 1. 0.01~0.03% of Vα24^+^ Vβ11^+^ NKT cells in peripheral blood from lung cancer patients 2. Lower NKT cells in recurrent patients compared to healthy controls (*p* < 0.01)	(158)

**Figure 2:**
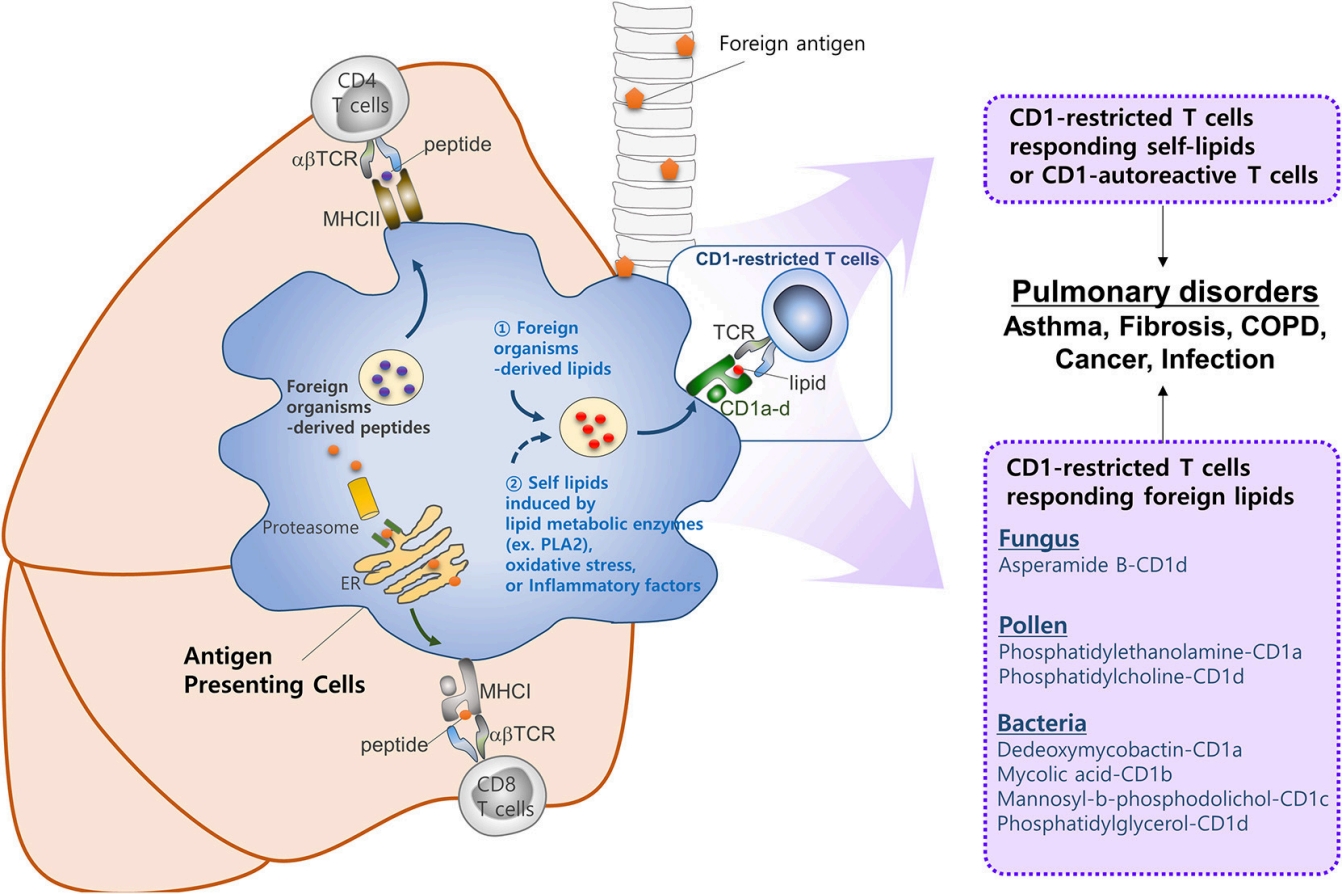
CD1-reactive T cells in pulmonary disorders. T cells play a critical role in pulmonary immune responses to both non-self antigens (such as microbiota and allergens) and self-antigens. In general, T cells are activated upon recognition of peptide antigens presented to the TCR by classical MHCI and MHCII molecules. However, increasing evidence suggests that T cells recognizing lipid antigens presented by CD1 molecules play a significant role in immune responses in the lung. CD1 molecules can display a broad range of lipid antigens derived from foreign organisms (e.g., fungi, pollen, and *Mycobacterium tuberculosis*). Oxidative stress or inflammatory factors released by host, or lipid metabolic enzymes (e.g., PLA2) derived from foreign organisms, might be responsible for activating CD1-restricted T cells in the lung. However, further studies are required to identify self-lipid antigens, the mechanisms by which such antigens are generated, the circumstances under which CD1-autoreactive T cells are activated, and the roles they play in lung disorders.

Despite the large body of work on CD1-restricted T-cell biology, many questions remain in this field, as follows. First, our understanding of how group 1 CD1-restricted T cells participate in lung diseases is limited. The recent development of transgenic mouse models expressing human CD1a, CD1b, or CD1c will improve this situation. Indeed, two studies based on these mice showed that they generate group 1 CD1-restricted T cell responses that are consistent with the responses of human T cell lines (129, 130). While these studies mainly showed that CD1-restricted T cells participate in skin inflammation, these group 1 CD1 transgenic mice may be useful for examining the *in vivo* roles of CD1-restricted T cells in other diseases. Second, no study has confirmed that autoreactive CD1-restricted T cells contribute to development of lung diseases. By contrast, several reports suggest that self-lipid-reactive T cells may participate in the pathology of psoriasis, which is an autoimmune skin disease (129, 165). Autoimmune pulmonary diseases are either primary autoimmune diseases or systemic autoimmune diseases that show pulmonary manifestations: the latter include systemic sclerosis, systemic lupus erythematosus, rheumatoid arthritis, and Sjogren's syndrome (166). Given that self-lipid-reactive T cells are associated with development of psoriatic skin, it is highly likely that there are self-lipid antigens that could promote the lung manifestations observed in systemic autoimmune diseases. In addition, lipid antigens in the lungs still need to be defined. An allergic reaction in the lungs can be induced by house dust mite and ozone, which activate CD1-restricted T cells (35, 89). These substances have the potential to generate a neoantigen for CD1-restricted T cells. For example, house dust mite contains phospholipase A2 (PLA2), which converts self-lipids into neoantigens. Similarly, it is speculated that ozone may chemically modify self-lipids to render antigenic properties against CD1. Notably, many lipid ligands of CD1c do not make direct contact with the TCR to trigger CD1-mediated autoreactivity (12). Further investigation is required to understand the circumstances in which CD1-autoreactive T cells are activated and how they are involved in lung disorders (Figure 2). Finally, the role of NKT cells in asthma should be re-evaluated. There is a large body of work on the role of CD1-restricted T cells in asthma, which is the most studied disease in this field. This work has provided ample evidence that CD1-restricted T cells, especially NKT cells, are involved in the pathology of asthma in mouse models. However, controversy about the role of these cells in asthmatic humans arose in the late 2000s when some studies reported that asthmatics have low percentages of NKT cells (98, 99, 101). It seems quite possible, for example, that NKT cells play an important role in a subset of patients who are allergic to lipids. This possibility is supported by the fact that the triggering antigen in patients with allergic asthma is very heterogeneous; these patients show distinct individual patterns of reactivity to diverse allergens (167). While the most common allergens in patients with asthma do not contain lipid components, it seems possible that at least some patients would be allergic to lipid components that can activate NKT cells.

This review summarizes current knowledge of CD1-restricted T cells, their specific lipid antigens, and their role in lung disease. Further research is likely to reveal hitherto unsuspected contributions to the pathogenesis of various lung diseases.

## Author contributions

SR, JP, JK, and HK wrote and revised the manuscript.

### Conflict of interest statement

The authors declare that the research was conducted in the absence of any commercial or financial relationships that could be construed as a potential conflict of interest.

## Abbreviations

MHC: major histocompatibility complex
TCR: T cell receptor
IFN: interferon
IL: interleukin
CD1: cluster of differentiation 1
COPD: chronic obstructive pulmonary disease
ER: endoplasmic reticulum
PBMC: peripheral blood mononuclear cell
hCD1Tg: human CD1 transgenic
Mtb: *Mycobacterium tuberculosis*
NKT: natural killer T cells
DP: double-positive
α-GalCer: α-galactosylceramide
iGb3: isoglobotrihexosylceramide
AHR: airway hyper-responsiveness
OVA: ovalbumin
BALF: bronchoalveolar lavage fluid
β2M: β2-microglobulin
MDSC: myeloid-derived suppressor cell
IAV: influenza A virus
IPF: idiopathic pulmonary fibrosis
TB: tuberculosis
BCG: Bacillus Calmette-Guérin
DC: dendritic cell
NSCLC: non-small cell lung cancer
SCLC: small cell lung cancer
ILC: innate lymphoid cell
FEV1: forced expiratory volume in one second
MIP: macrophage inflammatory protein
CTGF: connective tissue growth factor
TNF: tumor necrosis factor.
